# Supplementary figures and images for: Stenoparib, an inhibitor of cellular poly (ADP-ribose) polymerases (PARPs), blocks in vitro replication of SARS-CoV-2 variants
Source: PLoS One. 2022 Sep 14;17(9):e0272916. doi: 10.1371/journal.pone.0272916 (PMC9473406; doi:10.1371/journal.pone.0272916)

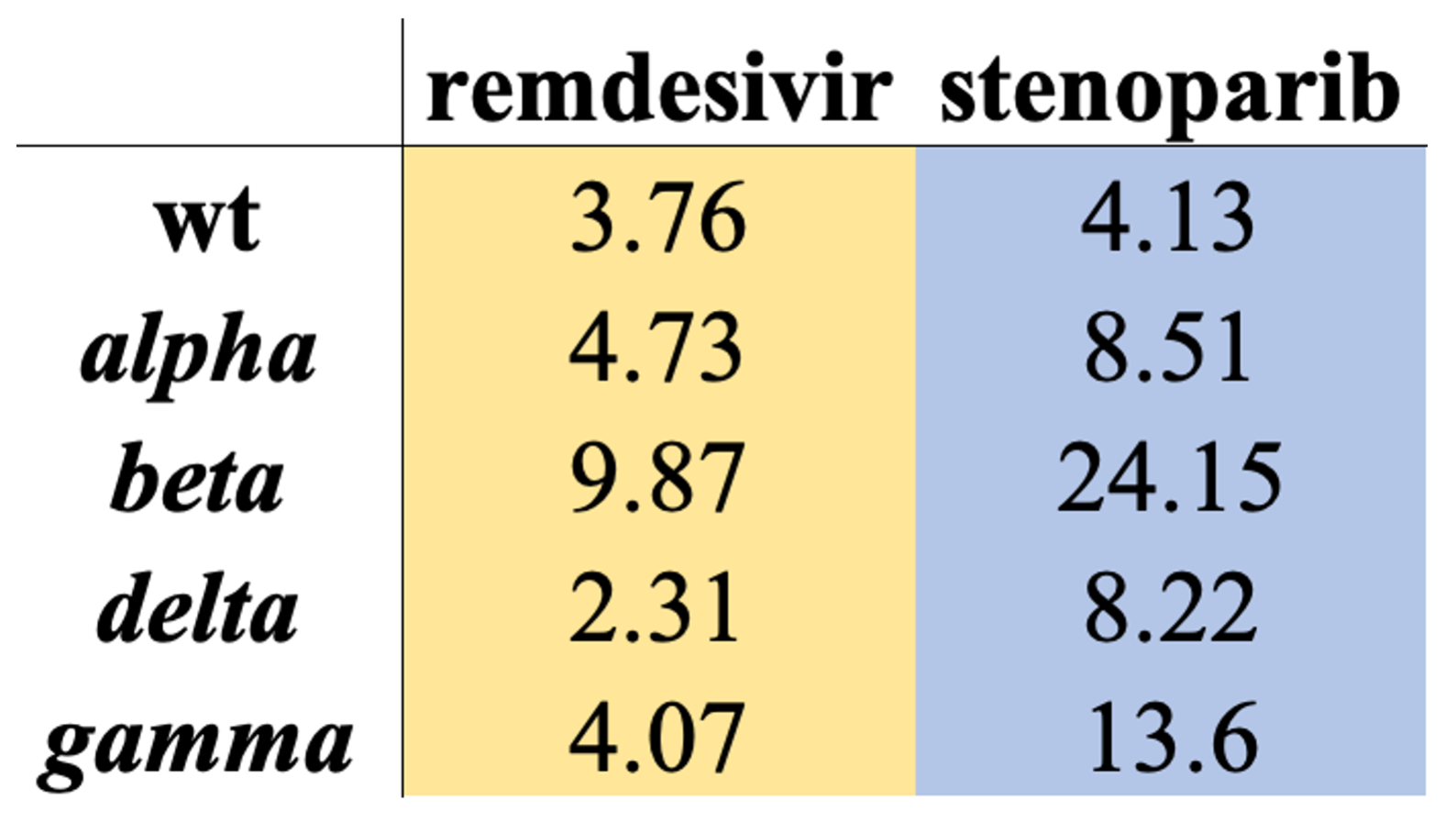

Supplement: S1 Fig — Stenoparib (gold shading) and remdesivir (blue shading) EC50 estimates for Vero E6 cells infected with SARS-CoV-2 wt, alpha, beta, delta, or gamma variants. (TIF) [file pone.0272916.s001.tif]

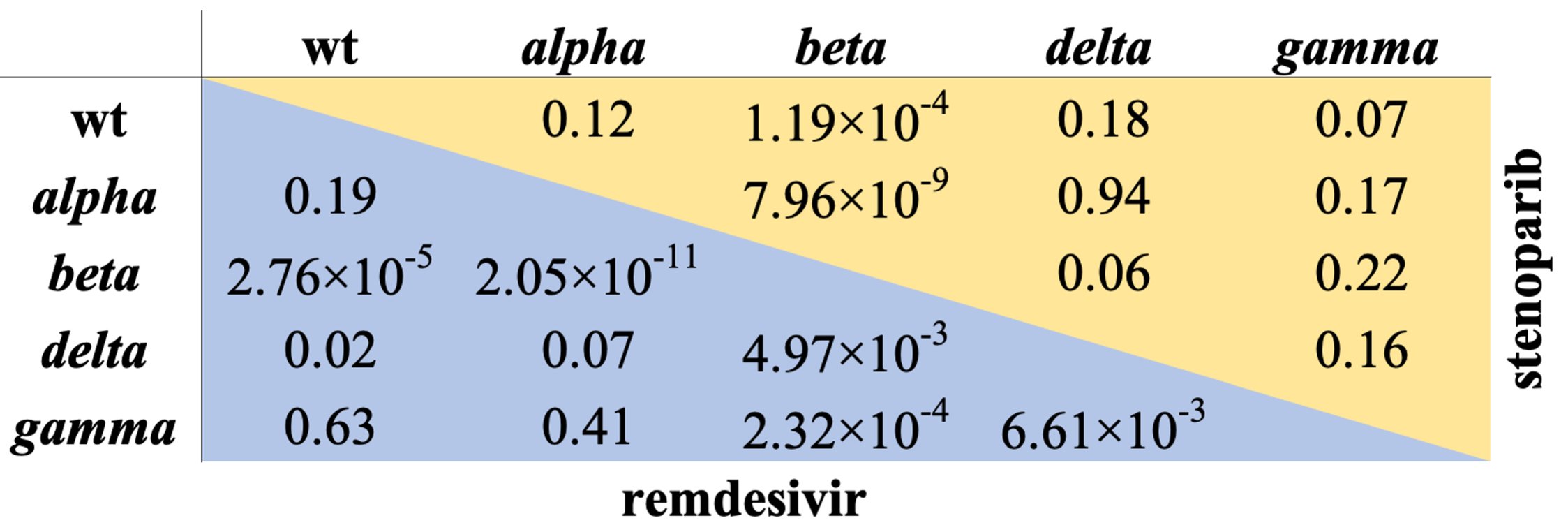

Supplement: S2 Fig — Stenoparib (above the diagonal, gold shading) and remdesivir (below the diagonal, blue shading) EC50 pairwise comparisons for SARS-CoV-2 wt, alpha, beta, delta, or gamma variants. (TIF) [file pone.0272916.s002.tif]
